# Lister’s Latest Antiseptic

**Published:** 1887-04

**Authors:** 


### Lister's Latest Antiseptic.

Sir Joseph Lister has recently substituted *sal-alembroth* for corrosive sublimate in antiseptic dressings. This salt has been long known, but has for many years fallen into neglect. It is a double salt obtained by subliming mercuric chloride with ammonium chloride, to which the formulæ $2NH_4Cl + HgCl_2 + H_2O$ has been assigned. It is more soluble in water than the mercuric chloride. To make any of the dressings antiseptic, all that is necessary is to soak them in this solution and dry. The salt not being volatile, does not require to be kept sealed in tin cases. Lister also colors these dressings with an aniline blue, 1-10000; the benefit to be derived from which is, that wherever an alkaline discharge comes in contact with the dressing the blue is removed and turned reddish, enabling one at once to see where the discharge has been, though the quantity was ever so small and had dried up before the dressing was removed. There is one precaution in using this dressing, and that is this; the dressing being dry and frequently handled, might have some septic matter from bedclothes, hands, etc., so he always dips it in 1-2000 corrosive sublimate chloride just before applying it. He is making a *sal-alembroth* protective, which will be surcharged with the antiseptic, so that, as a discharge comes through a dressing, it will come in contact with this protective and can be kept aseptic.—*The Polyclinic.*

ZONULAR CATARACT AND DENTAL MALFORMATIONS.—Dr. John B. Story cites nine cases, in addition to those collected by Horner, in which zonular cataract was associated with "rachitic" (not Hutchinson) teeth in children, who presented, generally, a history of infantile convulsions, cranial deformities, and defects in intelligence, all of which abnormalities he traced to the action of rickets. Horner's rachitic teeth are thus described: "They are, as a rule, thicker and coarser than the normal; the nearly formed incisor appears somewhat cubical and shapeless, though the shape can, in many cases, closely approximate the normal. The enamel, instead of gradually thinning away on the neck of the tooth, terminates abruptly in a swollen ridge. The delicate horizontal furrows to which the enamel owes its satin-like appearance become so enlarged as to even be visible to the naked eye, and sometimes, especially toward the cutting edge, a horizontal row of round holes marks the position of one of these excavated grooves. The body of the tooth terminates in a convex border at the cutting edge. The junction of the labial and lingual surfaces of the enamel runs as an irregular, zigzag line over the surface of the tooth. Sometimes the enamel is quite absent in grooves, the floor being formed by the discolored dentine."—*Ophthalmic Review.*

---

"In severe typhoid fever, characterized by slight variation between the morning and evening temperature, the secretion of a peptonizing gastric juice ceases, at least when the fever is highest. This is also the case in adynamic conditions, as in most cases of bilious pneumonia, in the later stages of malignant forms of dysentery, and of pneumonia occurring in infancy and old age. It requires, therefore, a violent attack, a condition of grave adynamia, very high and not too transient blood heat, to arrest the secretion of gastric juice capable of effecting the digestion of albuminous matter. A certain amount of variation occurs among different individuals. Thus, in some instances, instead of true gastric juice, there is secreted a fluid loaded with mucus of neutral or alkaline reaction, or if acid, rendered so by acetic or other adventitious acids.

"The important question now arises, how does it happen that, notwithstanding the gastric juice may in fever possess digestive power, there still exists a febrile dyspepsia in a marked degree? It has been contended that this is due to diminished secretion of hydrochloric acid. Without denying the appropriateness of supplying dilute hydrochloric acid when it is known to be deficient, the fact remains that its use under these circumstances seldom removes the indigestion."

